# Genotoxicity of Mercury and Its Derivatives Demonstrated In Vitro and In Vivo in Human Populations Studies. Systematic Review

**DOI:** 10.3390/toxics9120326

**Published:** 2021-12-01

**Authors:** Juana Sánchez-Alarcón, Mirta Milić, Lilia Patricia Bustamante-Montes, Keila Isaac-Olivé, Rafael Valencia-Quintana, Ninfa Ramírez-Durán

**Affiliations:** 1 Doctorado en Ciencias de la Salud, Facultad de Ciencias de la Conducta, Universidad Autónoma del Estado de México, Toluca 50180, Estado de México, Mexico; xcaretchava@hotmail.com; 2 Cuerpo Académico Ambiente y Genética UATLX-CA-223, Laboratorio “Rafael Villalobos-Pietrini” de Toxicología Genómica y Química Ambiental, Facultad de Agrobiología, Universidad Autónoma de Tlaxcala, Santa María Acuitlapilco 90120, Tlaxcala, Mexico; prvq2004@yahoo.com.mx; 3 Mutagenesis Unit, Institute for Medical Research and Occupational Health, 10000 Zagreb, Croatia; mmilic@imi.hr or; 4 Decanato de Ciencias de la Salud, Universidad Autónoma de Guadalajara, Guadalajara 451293, Jalisco, Mexico; patricia.bustamante@edu.uag.mx; 5 Facultad de Medicina, Universidad Autónoma del Estado de México, Toluca 50180, Estado de México, Mexico; kisaaco@uaemex.mx

**Keywords:** comet assay, chromosomal aberrations, sister chromatid exchange, micronucleus assay

## Abstract

Beside partial coverage in three reviews so far (1994, 2009, 2019), there is no review on genotoxic studies dealing with mercury (Hg) and human exposure using the most usual genotoxic assays: sister chromatid exchanges (SCE), chromosomal aberrations (CA), cytochalasin B blocked micronucleus assay (CBMN), and single-cell gel electrophoresis (SCGE or alkaline comet assay). Fifty years from the first Hg genotoxicity study and with the Minamata Convention in force, the genotoxic potential of Hg and its derivatives is still controversial. Considering these antecedents, we present this first systematic literature overview of genotoxic studies dealing with Hg and human exposure that used the standard genotoxic assays. To date, there is not sufficient evidence for Hg human carcinogen classification, so the new data collections can be of great help. A review was made of the studies available (those published before the end of October 2021 on PubMed or Web of Science in English or Spanish language) in the scientific literature dealing with genotoxic assays and human sample exposure ex vivo, in vivo, and in vitro. Results from a total of 66 articles selected are presented. Organic (o)Hg compounds were more toxic than inorganic and/or elemental ones, without ruling out that all represent a risk. The most studied inorganic (i)Hg compounds in populations exposed accidentally, occupationally, or iatrogenically, and/or in human cells, were Hg chloride and Hg nitrate and of the organic compounds, were methylmercury, thimerosal, methylmercury chloride, phenylmercuric acetate, and methylmercury hydroxide.

## 1. Introduction

Mercury (Hg) is a highly dangerous environmental pollutant, and many studies have evaluated the activity of Hg compounds in different test systems with a wide variety of biomarkers. Nevertheless, one that is striking is its possible genotoxic effect in human populations, even at low concentrations [1,2,3,4,5,6]. Some studies recognized mutagenic and teratogenic effects and reported that it can also induce cancer, with very scarce and controversial information about the mechanisms by which it induces such effects [7,8].

Hg can be found in air, water, and soil. Environmental Hg pollution is caused by natural phenomena (erosion, volcanic eruptions) and anthropogenic activities (metal smelting, industrial production). Due to the consumption of Hg-contaminated food, diverse populations have faced Hg-induced catastrophic diseases and mortality events [8].

Human beings may be occupationally or environmentally exposed to Hg or its inorganic and organic derivatives. Some of them, in addition to being persistent, belong to the most toxic substances known so far [7]. The predominant Hg toxic forms include: elemental Hg (Hg^0^); ionic Hg also called inorganic (i)Hg (II) or Hg^2+^; and organic (o)Hg, such as methylmercury (MeHg), classified as the most toxic among them [9].

Although the location of Hg’s discovery is not known in detail, it has been found in Egyptian tombs dating back to 1500 B.C., and was used by ancient Chinese, Greek and Roman populations for medical and cosmetic purposes. In the Middle Ages Hg became popular among alchemists who believed it was the “first metal” from which all others could be formed [10].

Hg that enters the marine environment comes from the atmosphere, in iHg form: Hg^0^, divalent (Hg^2+^) and particulate (Hgp). The elemental form is transformed into MeHg by the action of some bacteria. As those bacteria incorporate into the food chain (in/within aquatic organisms) and biomagnify, there is a possibility of greater Hg bioaccumulation. People are exposed to MeHg mainly through their diet, by eating contaminated fish and other foods [11,12,13]. Exposure to Hg^0^ or iHg can occur through inhalation during work activities, when Hg or its compounds are produced, used in processes, or incorporated into products. Occupational exposures have been reported in chlor-alkali, vinyl chloride monomer plants, Hg mines, small-scale gold and silver mining, refineries, manometer and thermometer factories, electrical switches, fluorescent lamp bulbs, dental clinics with inefficient handling, and the production practices of Hg-based products. Exposures can also occur due to the presence of dental amalgams; the use of some creams and soaps for skin lightening; as well as Hg presence in batteries; in some traditional medicines; from to its use in cultural practices, such as the use of thimerosal as a preservative in some vaccines or other pharmaceutical products; paints; and as an industrial catalyst. There is also exposure risk due to accidental spills or during natural processes (such as volcanic activity and the leaching of certain soils) [9,14].

Hg is a global pollutant that affects human health and ecosystems [15]. Factors that determine the occurrence and severity of adverse health effects include their chemical form; the dose; the age or developmental stage of the exposed person; and the duration and route of exposure (inhalation, ingestion and/or dermal contact). The main target organs for Hg toxicity are the nervous, renal, and cardiovascular systems, followed by the respiratory, gastrointestinal, hematologic, immune, endocrine, and reproductive systems; it is also involved in developmental disturbances [9,14,16,17,18,19,20].

Countries such as Japan, Iraq, Ghana, the Seychelles, and the Faroe Islands, have grappled with its effects, unraveling the insidious and debilitating nature of Hg poisoning [8].

Chronic exposure to low Hg levels due to global contamination/occupational risk has increased and concern has generated a series of investigations covering exposure sources, target organs, toxicity, and different metabolism, depending on the Hg type or species [15]. For example, Hg^0^ exposure occurs primarily from Hg^0^-based dental restorations [21]. Liquid Hg exposure is minimal, but the problem arises when it vaporizes; it can induce acute interstitial pneumonia when inhaled in high concentrations, or when absorbed into the blood where it damages the central nervous system.

To assess possible risks due to inhalation exposures, the United States Environmental Protection Agency (US EPA) established an inhalation reference concentration (RfC) for Hg^0^ of 0.3 μg/m^3^ in the air, based on the lowest observed adverse effectlevel (LOAEL) [9]. Reference doses (RfDs) have been established for mercuric chloride (HgCl_2_) of 0.3 μg/kg/day and MeHg 0.1 μg/kg/day. Several governments and other organizations have estimated tolerable weekly intake/reference levels for MeHg exposure that are intended to be protective against adverse effects from 0.7 to 1.4 μg MeHg/kg bw/week [9]. Due to fish consumption dominating MeHg exposure pathways for most of the human populations, many governments have provided recommendations or legal limits for the maximum allowable Hg amount and/or MeHg in fish to be sold on the market, with alimentary guideline levels from 0.2 mg MeHg/kg in non-predatory fish to 1 mg MeHg/kg in predatory fish [9,14]. Preliminary critical limits to prevent ecological effects due to Hg in organic soils have been set at 0.07–0.3 mg/kg for the total Hg soil content [14]. EPA’s reference MeHg dose is 0.1 μg/kg body weight per day according to the EPA’s Water Quality Criterion for the Protection of Human Health: Methylmercury [14]. Also, the Joint the Food and Agriculture Organization of the United Nations (FAO)/The World Health Organization (WHO) Expert Committee on Food Additives (JECFA) have established a MeHg provisional tolerable weekly intake (PTWI) of 3.3 μg/kg body weight per week [14]. Legislation also exists for limiting/prohibiting Hg in cosmetic products, stipulating that Hg and its compounds may not be present as ingredients in cosmetics, including soaps, lotions, shampoos, skin bleaching products etc. (except for phenyl mercuric salts for eye make-up conservation and eye-make-up removal products in concentrations not exceeding 0.007 percent weight to weight) [14].

Although systematic research on heavy metal exposure and effects started centuries ago, new discoveries, and large gaps in understanding the mechanisms and the right assessment of the health effects caused by environmental and occupational human exposure are still pointing out the necessity for new data collection and further research. Beside partial coverage in three reviews so far (1994, 2009, 2019) [1,7,22], there is no literature review of the genotoxic studies dealing with Hg, its derivatives and human exposure using the most usual genotoxic assays: sister chromatid exchanges (SCE), chromosomal aberrations (CA), cytochalasin B blocked (CBMN), and single-cell gel electrophoresis (SCGE or alkaline comet) assays, making this review paper a valuable contribution to this field.

## 2. Materials and Methods

The Pubmed and Web of Science database (Indexed as also SCI-EXPANDED, SSCI, A&HCI, CPCI-S, CPCI-SSH, BKCI-S, BKCI-SSH, ESCI, CCR-EXPANDED, IC.) were searched for all years, with the last date of search being 23 October 2021. The retrieved articles were searched both in English and in Spanish.

Pubmed was searched with these terms: “mercurial”(All Fields) OR “mercurials”(All Fields) OR “mercuries”(All Fields) OR “mercury poisoning”(MeSH Terms) OR-“mercury” (All Fields) AND “poisoning” (All Fields)-OR “mercury poisoning” (All Fields) OR “mercurialism” (All Fields) OR “mercury” (MeSH Terms) OR “mercury” (All Fields) OR “mercury s” (All Fields), and then in combination with:

Sister chromatid exchange: AND-“sister chromatid exchange” (MeSH Terms) OR-“sister” (All Fields) AND “chromatid” (All Fields) AND “exchange” (All Fields)-OR “sister chromatid exchange” (All Fields)-(revealing, in total, 790 articles);

Chromosomal aberration: AND-“chromosome aberrations” (MeSH Terms) OR-“chromosome” (All Fields) AND “aberrations” (All Fields)-OR “chromosome aberrations” (All Fields) OR-“chromosomal” (All Fields) AND “aberration” (All Fields)-OR “chromosomal aberration” (All Fields)-, (revealing in total 128 articles);

Micronucleus: AND “micronucleus” (All Fields) OR “micronuclei” (All Fields) (revealing, in total, 62 articles);

Comet assay: AND-“comet assay” (MeSH Terms) OR-“comet” (All Fields) AND “assay” (All Fields)-OR “comet assay” (All Fields)-(revealing in total 60 articles);

Web of Science was searched with ALL FIELDS checked, with the words mercury and in combination with either comet assay (in total 105 articles), micronucleus (in total 142 articles), sister chromatid exchange (in total 33 articles) and chromosomal aberrations (in total 50 articles).

The results were reviewed and only those with data evaluating genotoxicity and related biomarkers were selected. After duplicate references were eliminated, 66 articles dealing with human samples in vitro or in vivo were selected in relation to the analysis of genotoxicity. This article aims to provide an overview of the available data in this regard, covering the results obtained with Hg and its compounds in human short-term in-vitro test systems, as well as the cytogenetic data that have emerged from the biomonitoring of exposed individuals. The Preferred Reporting Items for Systematic Reviews (PRISMA) checklist [Tables S1 and S2], and the flow diagram for the selection of studies [Figure S1], were completed.

## 3. Results

### 3.1. In-Vitro Genotoxic Effects for Inorganic (i)Hg Compounds

iHg compounds or salts, such as mercury sulfide (HgS), mercury oxide (HgO), and HgCl_2_, are found as powders or crystals, and the natural mineral cinnabar, with the highest Hg content in the form of Hg sulfide (HgS), can be formed by Hg^0^ vapor metabolism (Hg^0^ biological oxidation) or by MeHg metabolism (MeHg demethylation by the intestinal microflora activity) [23].

iHg (for example, ammoniated Hg) is a common but dangerous ingredient found in skin lightening creams and soaps [24,25]. Many such products contain Hg levels higher than the limit established in the Minamata Convention on Mercury of 1 mg/kg (1 ppm). Despite having been banned in many countries, Hg-containing skin lightening products are often easily obtainable [26].

iHg does not cross the placental or blood-brain barriers; however, it can be found in the brain of neonates, due to the absence of a fully formed blood-brain barrier [27]. The elimination iHg half-life is approximately 1–2 months (depending on the compound) [28]. Table 1 lists the studies on genotoxic in-vitro effects by iHg compounds [29,30,31,32,33,34,35,36,37,38,39,40,41,42,43].

#### 3.1.1. Mercury Chloride (HgCl_2_)

HgCl_2_ was able to induce CA in peripheral blood lymphocytes (PBL) [31] and SCE in white blood cells [30]. However, negative results at sub-toxic concentrations have been previously reported with the CA test [29]. Bérces et al. (1993), using the MN assay, detected the genotoxic effects of metal ions, including Hg [32]. A linear increase in the MN frequency was observed with increasing HgCl_2_ concentration. The CA in human lymphocytes and the levels of 8-hydroxy-2’-deoxyguanosine (8-OHdG) in mononuclear cells (PBMC), exposed to different concentrations of this compound, increased in a concentration-dependent manner and were significantly higher than those found in controls [33]. However, the results of the analysis of the frequency of hypoxanthine-guanine-phosphoribosyl-transferase (HPRT) mutants in the human lymphoblastoid thymidine kinase heterozygote (TK6) cell line, after exposure, were inconsistent with respect to their mutagenic effects, although they exhibited clear cytotoxic effects [34].

The damage produced to DNA by HgCl_2_ has been evaluated in a human fetal liver cell line (WRL-68), using the comet assay [35]. The compound was able to induce single strand breaks or alkali sensitive sites. The percentage of damaged nuclei and the average comet tail length (TL) of DNA increased as the concentration and exposure time increased. Recovery time from DNA damage was 8 h after partially removing the metal with PBS-EGTA [35]. A concentration-dependent comet formation was induced in U-937 cells, with concentrations between 1 and 5 µM [36]. In human leukocyte cultures, no effects were found on cell proliferation kinetics, however, the SCE frequency, as well as the C-anaphases frequency, were significantly higher with respect to the values found in the control [37].

Genotoxicity of HgCl_2_ was assessed by CA and polyploidic cells count [38]. A significant increase of gaps and breaks was found at 0.1 and 1000 µg/L, but HgCl_2_ did not induce aneuploidies at any concentration tested. Rozgaj et al. (2006), in a preliminary study with the purpose of HgCl_2_ genotoxicity evaluation in cultured human lymphocytes using CBMN assay, did not find significant differences between the samples analyzed after 24 h of exposure, but, after 48 h, they found a significant increase in MN frequency, although not in a concentration-dependent manner [39]. However, inconsistent results were found by the same research group using the SCGE assay, evaluating the TL, tail moment (TM) and tail intensity (TI), with HgCl_2_ concentrations of 50, 100, and 200 µM [40]. At 24 h exposure to doses of 50 and 100 µM, TL values were statistically different when compared with controls, but no significant differences were found in the TM or TI. At 48 h exposure, they found significant differences in TL between the positive control and the other samples, and the control sample, the highest concentration of which was 200 µM. For TI and TM, only the positive control sample demonstrated significant difference from all other samples.

Damage to DNA in human lymphocytes and the cells of salivary gland tissue was evaluated by analyzing DNA migration due to single-strand breaks, alkali-labile sites, and incomplete excision repair using the SCGE assay [41]. Genotoxic effects were evaluated below a cytotoxic dose level, with a significant increase in dose-dependent DNA migration demonstrated after exposure to 2.5 µM HgCl_2_ and above, in both test systems, when compared with the negative control. On the other hand, in TK6 cells, there was a dose-dependent increase in OTM from 0.1 mM and up to 2 mM [42].

#### 3.1.2. Mercury Nitrate

Only one study was found regarding the genotoxic potential of Hg nitrate, reporting no SCE induction in cultured human lymphocytes; however, at 30 µM, Hg nitrate could produce endoreduplication (E) [43].

### 3.2. In-Vitro Genotoxic Effects for Organic Compounds

Among the organic Hg compounds, one of greatest concern is MeHg (CH_3_Hg), produced in the environment mostly by microorganisms (bacteria and fungi), rather than by human activity.

CH_3_Hg is widely distributed in the body, crossing both blood–brain and placental barriers in humans [44]. The main system affected by CH_3_Hg is the central nervous system, although others, such as urinary and immune systems are also compromised. From the point of view of its genotoxic potential, it has been the most evaluated species.

Ethylmercury (C_2_H_5_HgCl) and phenylmercury (C_6_H_5_HgCl) chloride were able to induce chromosomal alterations in human HeLa S3 cell cultures [45], and phenylmercury acetate (PMA or CH_3_COOHgC_6_H_5_) increased the SCE and E frequency in a concentration-dependent manner in cultured human lymphocytes [43].

The same situation occurred for dimethylmercury [(CH_3_)_2_Hg], which could induce numerical and structural CA, although with a lower potency compared with the effect induced by methyl chloride in human lymphocytes [46].

A similar effect was found with thimerosal, also known as thiomersal, or with the trade names merthiolate, mertodol or metorgan. This oHg compound, o-carboxyphenyl-thio-ethyl-sodium salt, with a recognized antiseptic and antifungal action, is used especially in vaccines in domestic and farmed animals and in humans. The WHO considers small doses of thimerosal safe regardless of multiple/repetitive exposures to vaccines that are predominantly taken during pregnancy or infancy. In human lymphocytes a significant MN induction was observed in the CBMN test [3], and there was also s significant increase in SCE assay with/without metabolic S9 activation, together with a significant decrease in mitotic (MI) and proliferation indexes (PRI) [47]. There are no other studies performed on human samples with genotoxic assays of our interest, but since its main metabolised compound is C_2_H_5_HgCl, and exposure is usually in the early developmental stages of organism, toxicological assays were performed, and although in-vitro and in-vivo conditions did not give similar results, and, while the exact mechanisms of action are still unknown, it has been demonstrated that it causes immunotoxic and neurotoxic effects [48]. These data raise some concern about the widespread use of thimerosal in some vaccines that are still in use, mostly in developing countries.

#### Methylmercury (MeHg or CH_3_Hg)

CH_3_Hg induces structural and numerical CAs in human lymphocytes [49]. Likewise, it increases the SCE frequency in lymphocytes from whole blood cultures [30,49], producing chromosome breaks [50] and altering their segregation [31], in the same test system.

Ogura et al. (1996) reported that it induced a significant concentration-dependent increase in CA in human lymphocytes and in the levels of 8-hydroxy-2’-deoxyguanosine (8-OHdG) in PBMC, being more potent than the inorganic methyl chloride species [33]. Similarly, the frequency of SCE and E increased to 20 µM, in the same test system [43].

Studies on this compound have demonstrated inconsistencies regarding its mutagenic capacity when analysing the hypoxanthine phosphoribosyltransferase (hprt) locus mutations frequencies and CA number in the TK6 cell line, also demonstrating cytotoxic properties and causing a marked mitosis frequency reduction, alone or in combination with HgCl_2_ [34].

Its genotoxicity was demonstrated in human PBL, through CA and polyploid cells’ induction; as proof of its cytotoxicity, it lowered the mitotic index (MI) [38,51].

A significant increase in the frequency of both genotoxicity biomarkers and a significant MI decrease was observed at all concentrations evaluated compared to the control, either alone or in an evident synergistic combination with HgCl_2_.

Human brain cell lines of glioblastoma (U373) and neuroblastoma (B103) were exposed to methylmercury chloride (CH_3_HgCl), for 24 (U373) or 48 (B103) hours [6]. The binucleation index, the frequency of cells with MN, as well as the metaphasic MN and nucleoplasmic bridges were determined. Statistical analysis showed significant increases and percentages in the treated cells. Each cell line was shown to be different from each genotoxic damage biomarker, which seems to indicate the existence of different toxicity mechanisms. This work demonstrated the ability of CH_3_HgCl to cause genotoxicity in cells of the nervous system, with relatively low levels of exposure. In a human lymphoblastoid cell line (TK6) an increment of OTM was induced [42].

Crespo-López et al. (2016) analysed the possible genotoxicity and alterations in the cell cycle and proliferation of a glioma line (C6) exposed to a low, non-lethal and non-apoptotic concentration of CH_3_HgCl [52]. Treatment without promoting cell death significantly increased genotoxicity markers (DNA fragmentation, MN, nucleoplasmic bridges, and nuclear buds). In the same way, it caused changes in the cell cycle, which suggests cell cycle arrest. This work demonstrated that exposure to a sublethal CH_3_HgCl concentration, considered relatively safe, according to current limits, induces genotoxicity and alterations in the proliferation of cells of glial origin.

Patnaik and Padhy (2018) compared the genotoxicity of CH_3_HgCl and methylmercury hydroxide (CH_3_HgOH) in the human neuroblastoma cell line SH-SY5Y using the comet assay and demonstrated that both compounds were capable of inducing DNA fragmentation, with CH_3_HgCl being the most toxic for this cell line [53]. Table 2 lists the studies on the in-vitro genotoxic effects of oHg compounds.

### 3.3. Genotoxic Effects in Exposed Individuals

Human populations can be exposed to Hg^0^ or its derivatives accidentally, through occupational exposure, or through food. The first in-vivo studies on the genotoxic effects induced by exposure to this metal were carried out during the 1970s and 1980s, determining the CA, MN, and SCE frequencies. The results of epidemiological studies related to human Hg exposure have been increasing since the end of the last century, which makes the results more reliable, demonstrating an increase in genotoxicity in human populations exposed to Hg through diet, the occupation or wearing of dental fillings [22]. Table 3 represents studies dealing with genotoxic effects in human populations exposed to Hg.

#### 3.3.1. Accidental Exposures

Mudry de Pargament et al. (1987) reported the induction of SCE in lymphocytes of children exposed to PMA used in diaper disinfection [60].

#### 3.3.2. Exposure from Contaminated Food

The more frequent consumption of seal meat (six times a week, an average diet Hg concentration of 62.5 µ/L) increased SCE frequency when compared to less frequent consumers (once or twice a week, an average diet Hg concentration of 22 µ/L) [65].

However, Monsalve and Chiappe (1987) did not find a differences in SCE or CA frequency between subjects exposed to this compound through the consumption of fish caught in a contaminated area and subjects who consumed fish from uncontaminated area, even though the difference in hair samples CH_3_Hg concentrations between those two groups was statistically significant and higher in the subjects who ate fish from the area [56].

Skerfving et al. (1970) compared CA frequency in two groups of individuals in relation to the consumption of fish contaminated (or not) with CH_3_Hg and found a correlation of Hg concentration in erythrocytes, with fequent structural chromosomal rearrangements, but not with polyploidy or aneuploidy [54]. Later, in an extension of their study, they found a slight increase in CA frequency [55].

#### 3.3.3. Occupational or Environmental Exposure

Verschaeve et al. (1976) found an increase in aneuploidy frequency in subjects exposed to metallic (m)Hg, amalgams, C_6_H_5_Hg, and C_2_H_5_Hg^+^ [58]. Exposure to the latter also showed an increase in the structural CAs frequency, PMA induced a significant increase in hyperpolyploidy frequency [59]. Subjects with Hg amalgams showed an increase in aneuploidy frequency when compared with the control group [79].

Contrary that reported in these studies, the same research group (Verschaeve et al. 1979) and Mabille et al. (1984), found no genotoxic effects in workers exposed to metallic (m) Hg [61,62]. As in the latter cases, in a group of workers exposed to mHg vapors, as well as in another group exposed to a mixture of CH_3_HgCl and C_2_H_5_HgCl, there was no significant increase in genotoxic damage, but, when combining the two groups, there was a significant increase in the frequency of acentric fragments.

Popescu et al. (1979) determined the genotoxic damage due to occupational exposure to mHg or oHg [64]. Exposed workers had higher CA incidence when compared to the control group, but with no differences observed between the unexposed and exposed groups with respect to the frequency of aneuploidies and polyploidies.

A significant SCE increase was found in workers in two factories exposed to mHg and iHg [66]. Using the MN test, Barregård et al. (1991) did not find an increase in genetic damage in workers exposed to a chlor-alkali plant when compared with a control group [67]. On the other hand, fulminant Hg-exposed workers in an explosives factory presented a statistically significant increase in MN and CA frequency when compared with controls but found no correlation between this type of damage and Hg urine concentrations [73].

In Slovenian miners, a significant MN, CA, and SCE frequency increase was related to their employment seniority [74]. Similar results were found by Cruz-Esquivel et al. (2019) using the comet assay in peripheral blood lymphocytes and a micronucleus (MN) cytome assay (BMCyt) in exfoliated buccal cells of Colombian miners [78].

Higher levels of CA, MN, SCE, and HGPRT mutations were found in workers of a chloroalkali plant [69], a mercury-producing plant [70], a battery plant and dentists [71]. In contrast, workers in a chloroalkali plant exposed to Hg vapor did not show significant differences in CA and MN frequencies in peripheral lymphocytes [68], nor were MN or SCE different in harbour workers with potential exposure to river silt aerosols [72].

Cytogenetic monitoring of fishermen with environmental Hg exposure was carried out by evaluating the MN, CA, and SCE frequencies in PBL [75]. A statistical correlation was found between the MN frequency and the total Hg blood concentration, supporting the usefulness of this biomarker for early DNA damage detection. In the same way Queiroz et al. (1999) observed a significant increase in the MN percentage of people exposed to Hg compared to an unexposed group [70]. No correlation was found between the MN percentage and age, duration of exposure, or urinary Hg concentrations with the levels considered biologically safe for the exposed population.

Amorim et al. (2000) examined the cytogenetic alterations in the peripheral lymphocytes of a population who lived on the banks of the Tapajós River in Brazil, with respect to contamination by CH_3_HgCl, using hair Hg as a biological indicator of exposure [2]. The results showed a clear relationship between CH_3_HgCl contamination and cytogenetic damage in lymphocytes at levels well below 50 mg/g—the values considered safe by the World Health Organization (WHO). The main changes found consisted of polyploidies, chromatid breaks and an MI decrease.

Cebulska-Wasilewska et al. (2005) carried out a population study to evaluate whether occupational Hg exposure can cause genotoxicity and affect the efficacy of DNA repair mechanisms [4]. Although the exposure did not generate differences when the SCE test was used with respect to the control individuals, the chromosomal damage detected by the comet test increased significantly in the lymphocytes of the exposed workers, in the same way, as the authors determined, that Hg causes a significant decrease of DNA repair capacity. The latter could eventually lead to a carcinogenic process [84]. In this regard, the International Agency for Research on Cancer (IARC 1993, WHO) classifies CH_3_Hg within group 2B, as a possible carcinogen, with other inorganic forms classified in group 3 (“It cannot be classified with respect to its carcinogenicity to humans”) [85]. Currently there is still no evidence that either CH_3_Hg or other iHg forms can cause cancer in humans.

In their study on cytogenotoxicity in uroepithelial cells from women exposed to inorganic Hg in a mining area, Soto-Ríos et al. (2010) provided evidence that these people are at increased risk of developing not only different types of DNA damage, but, are also at increased risk of tumour development [76]. The first cellular changes that can increase the possibility of cancer risk indicate that exposure to Hg-containing mining wastes is a health risk, recommending that these cellular changes should be considered in the preliminary assessment of the health risks associated with this occupational exposure.

As a part of the BioMadrid project, derived from the concern for DNA stability in newborns and their parents who were environmentally exposed to various metals, an association was found between elevated Hg blood levels in fathers with an increase in the frequency of CBMN, which were both significantly higher when compared to the fathers who had “normal” Hg levels. The results showed a statistically significant correlation in the frequency of CBMN parameters between parents and new borns. An association was found also between the CBMN rate and elevated Hg levels in mothers and fathers, but not in newborns. This result provides information on the relationship between shared and genetic environmental effects [77].

Crespo-López et al. (2011), analysed, in vitro, the Hg blood genotoxicity in the lymphocytes of Amazonian individuals using two methods, MN and CA [51]. The induced frequencies were very low and only the cell cycle was significantly inhibited when comparing exposed versus unexposed populations.

The evaluation of genotoxic damage in artisanal mining workers exposed to Hg was carried out by Rosales-Rimanche et al. (2013) [57]. It was reported that 15% of the workers presented MN in buccal epithelium cells, also registering other nuclear abnormalities such as nucleoplasmic bridges, buds, and binucleated cells, demonstrating the genetic damage association with occupational exposure.

Castaño Arias et al. (2014) determined the magnitude of genetic damage with Hg exposure in mining workers using the comet test, finding greater damage (TI values) in the exposed group when compared with the control group [82]. Similarly, in a heavy metals-effects study in a human population of the La Mojana region, Colombia, Calao and Marrugo (2015) found significant associations between Hg presence and DNA damage in the same bioassay and using the same biomarker [63].

#### 3.3.4. Amalgams

Dental fillings provide significant iatrogenic exposure to xenobiotic compounds. Experimental data suggest that amalgams, which contain Hg, cause a deterioration of the pro-antioxidant cellular redox balance. A study was carried out to evaluate the potential genotoxicity of dental restorative compounds in the PBL of exposed subjects compared with a control group, using an SCGE assay [80]. The comet TL, TI, TM, and Olive tail moment (OTM) were twice as high in the exposed group with significant differences from the unexposed group. In addition, the authors demonstrated the association between the number of amalgams and the exposure time with DNA damage. The main mechanism underlying genotoxicity was attributed to the ability of implants (mercury) to trigger the generation of reactive oxygen species (ROS), capable of causing DNA damage [80].

Genotoxic damage in the oral mucosa cells of subjects with Hg-based dental restorations (amalgams) was evaluated with SCGE, MN, and a nuclear abnormalities (NA) test as markers of cell death. The results showed that amalgams can induce genetic damage by increasing MN frequency and marginally by the damage detected through the comet assay. The relevance of this study lies in the fact that subjects with Hg-based restorative materials are exposed continuously and for long periods of time to this metal [81].

Hg dental amalgam has a long history of apparently safe use despite the continuous release of mercury vapor. However, some studies suggest that it can cause DNA damage, particularly for individuals with common genetic variants [86]. This and other studies suggest that susceptibility to Hg toxicity differs between individuals based on multiple genes, so exposure levels to Hg vapor from dental amalgams can be dangerous for certain subpopulations. For this reason, efforts are being made to reduce or eliminate the use of Hg-based dental amalgam.

Similar results were obtained by Mary et al. (2018) using MN test in the oral mucosa cells [83]. The analysis of the alterations was carried out in the same subject before and after dental restoration with amalgams, serving as their own controls. The mucosa samples were taken before the intervention and 10 days after. A statistically significant difference was found in MN frequencies when comparing both samples (before and after). Similarly, the damage increased as the number of restorations in the individual increased.

## 4. Compounds against Hg Genotoxicity

Efforts have been made to find therapeutic agents capable of reducing the genotoxicity of different natural or anthropogenic compounds. As can be seen in this review, the genotoxicity induced by Hg compounds remains controversial. However, different agents have been tested in order to assess their antigenotoxic (or protective) properties in relation to the effects induced by Hg and its derivatives [37]. Epidemiological studies have shown that enzyme activity is altered in the exposed populations, which could contribute to an increase in genotoxic damage, since it has been proposed that Hg can inhibit antioxidant enzyme activity causing stress within the cells and organism [87]. Thus, the analysis of these markers could be useful in the evaluation of compounds’ toxicities [22].

Catalase (CA) and superoxide dismutase (SOD) have been used for their antigenotoxic (protective) properties against PMA effects, however, at concentrations of 75 and 150 g/mL, they were not able to present this property [43]. L-ascorbic acid (vitamin C) has demonstrated its protective capacity against CH_3_HgCl-induced damage in cultured human leukocytes, probably due to its antioxidant and nucleophilic nature [37].

Purohit and Rao. (2014) evaluated the protective effect of melatonin (MLT) and α-tocopherol against Hg-induced genotoxicity in cultured human lymphocytes using the SCE test, cell proliferation and the replication index [88]. Exposure to the metal significantly increased the SCE frequency, cell proliferation kinetics inhibition and caused a decrease in the replication index, compared to controls. The addition of α-tocopherol and MLT individually and in combination showed a mitigating effect by reducing the genotoxic potential in the treated cultures. The percentage of improvement was comparatively high with both MLT and α-tocopherol, but also with their combination. Similar results were found when melatonin, curcumin, and andrographolide were evaluated against the Hg genotoxic effect in the same test system and with similar biomarkers. The results revealed a CH_3_HgCl dose-dependent increase with the SCE test after Hg treatment, while supplementation with these three antioxidant compounds effectively abrogated genotoxic damage in treated cultures and improved cell cycle kinetics. The antimutagenic activity of these compounds on Hg-induced genotoxicity was in the following order: melatonin > curcumin > andrographolide [89].

Ali (2018) evaluated the role of garlic and vitamin E in mitigating the genotoxic damage induced by CH_3_HgCl in human lung cells line WI-38 [90]. The treatments led to a significant and dramatic increase in DNA damage, evidenced by the comet TL and TM values when compared with the control samples’ values. However, garlic and/or vitamin E significantly reduced DNA damage in these treated cells. Protection with garlic alone was more effective than with vitamin E alone, while the combination of both was the most effective regimen.

## 5. Mechanism of Hg Genotoxic Action

Like other metals, Hg damages DNA through multiple mechanisms, recognizing its ability to bind to sulfhydryl groups. Different hypotheses have been raised about the possible molecular mechanisms of Hg genotoxicity, involving four main processes that lead to genotoxicity: the generation of free radicals and oxidative stress, action on microtubules, influence on DNA repair mechanisms, and direct interaction with DNA molecules [7,22].

The greatest contribution to the genotoxicity of Hg and its derivatives is due to their ability to generate ROS species, accompanied by the decrease in protective glutathione reserves. The genotoxic capacities of the different species are qualitatively comparable, which may suggest a differential bioavailability and the participation of a common genotoxic entity [1]. ROS are formed when Hg enters the cell directly through the plasma membrane or through protein transporters [37,43,80].

The damage can be direct, by oxidizing nitrogenous bases, or indirect, by interacting with other biologically important molecules, such as fatty acids, DNA polymerases, and microtubules [4,77,81,85,87,91]. Glutathione levels, a potent free radical scavenger and metal chelating agent, have been reported as decreased in Hg-exposed populations [2,87].

Cebulska-Wasilewska et al. (2005) speculated that occupational exposure to low Hg concentrations, within the permitted ranges, interferes with DNA repair processes by recombination and by base-pair cleavage [4].

## 6. Conclusions

The versatility of Hg compounds explains their many applications in various areas of industry. Its growing use has resulted in a significant increase in environmental pollution and in the increased number of episodes of human intoxication, arousing the concern of international organizations. However, the consequences of these poisoning outbreaks are not yet fully understood, especially when we consider the long-term effects of chronic exposure at relatively low levels [22].

The genotoxic effect caused by Hg still generates great controversy, given the diversity of responses that different Hg compounds can produce. CH_3_Hg compounds stand out as the most genotoxic among the diversity of forms derived from this metal [5,22].

Genotoxic alterations, such as CA and MN, have been detected in populations chronically exposed to Hg levels below the safety values defined by the WHO [2,5,80], using mainly peripheral blood cultures and, in some cases, oral mucosa epithelial cells.

The findings of several studies aimed at the biomonitoring of cytogenetic effects on PBL from people exposed to Hg and its compounds from accidental, occupational, or food sources were negative, controversial, doubtful, or uncertain as to the actual role played by Hg in some positive results [1,5,22]. The discrepancies found may be due to the different potency of the iHg and oHg derivatives, as well as to the different protocols applied in terms of exposure times, bioassays, and biomarkers, due to their different sensitivities. It would be pertinent to standardize genotoxicity tests, in order to have more reliable results.

## Figures and Tables

**Table 1:** Studies on in vitro genotoxic effects by inorganic Hg compounds.

Compound	Cell Type	Assay	Concentrations	Results	References
mercury chloride (HgCl_2_)	L	CA	<3.0 × 10^−8^ M	No significant differences (*p* > 0.05)	Paton and Allison 1972 [29]
	WB	SCE	8 × 10^−8^ M–2.5 × 10^−4^ M	Dose-dependent increase from 4 × 10^−7^ M, 10.57 ± 0.55 SCE/cell (*p* < 0.05) up to 5 × 10^−5^ M, 16.54 ± 0.69 SCE/cell (*p* < 0.001) vs. 8.86 SCE/cell in control	Morimoto et al. 1982 [30]
	Ly	CA	1–150 µM	Significant increase from 50 µM, with 5.00% of chromatid- or chromosome-type aberrations (*p* < 0.05) up to 150 µM with 7.00% of chromatid-type aberrations (*p* < 0.01) vs. 1.67% of chromatid- or chromosome-type aberrations in the control, unrelated to increased concentration.	Verschaeve et al. 1985 [31]
	WB	MN	10^−3^–10^−1^ M	A linear increase in MN frequency.	Bérces et al. 1993 [32]
	Ly	CA	2–50 µM	Dose-dependent increase from 5 × 10^−6^ M with 7.3 ± 0.9 CA (*p* < 0.05) up to 20 × 10^−6^ M with 14.3 ± 0.9 CA (*p* < 0.001) vs. 2.7 ± 1.2 in the control.	Ogura et al. 1996 [33]
		MN		Significant increase at 20 × 10^−6^ and 50 × 10^−6^ M with 43 and 65 cells with MN cells respectively (*p* < 0.001) vs. 25 cells with MN in the control in 3000 cells.	
		8-OHdG	5–20 µM	Significant increase of 8-OHdG levels at 10 × 10^−6^ M (1.047 ± 0.202) and 20 × 10^−6^ M (2.091 ± 0.539) (*p* < 0.05) vs. 0.394 ± 0.144 in the control.	
	TK6	CA	10–2000 ppb	No significant differences (*p* > 0.05)	Bahia et al. 1999 [34]
		HPRT	0.1–1000 ppb		
	WRL-68	SCGE	0.5 µM, 5 µM	Significant differences (*p* < 0.05) at 0.5 × 10^−6^ M (TL 43.4 ± 2.1 µm) and 5 × 10^−6^ M (TL 69.6 ± 0.7 µm) vs. TL 31.7 ± 1.6 in the control with 3 h treatment. TL 74.4 ± 0.7 was induced with 7 h treatment (*p* < 0.05)	Bucio et al. 1999 [35]
	U-937	SCGE	1–50 µM	With 5 µM mean TL at 24 h was 5.5 ± 0.06 mm; at 48 h, 7.2 ± 0.06 mm; and at 72 h, 8.9 ± 0.04 mm.	Ben-Ozer et al. 2000 [36]
	L	SCE	1.052, 5.262 and 10.524 µM	Significant increase for lowest (*p* < 0.5) and higher concentration (*p* < 0.001) with 6.382 ± 0.067 and 8.732 ± 0.111 respectivelly vs. 5.747 ± 0.110 in the control	Rao et al. 2001 [37]
	L	CA		Significant increase at higher concentrations for C-anaphases (*p* < 0.001) with mean values of 2.75 ± 0.25 and 3.75 ± 0.40 vs. 1.00 ± 0.00 in the control	
	Ly	CA	0.1–1000 μg/L	Significant gaps and breaks increase (*p* < 0.5) at 0.1 (1.4%) and 1000 (1.3%) µg/L vs. 0.7% in the control	Silva-Pereira et al. 2005 [38]
	Ly	CBMN	10, 50, 100, and 200 mM	24 h exposure- no significant difference. 48 h- no dose-related-MN frequency increase	Rozgaj et al. 2006 [39]
	WB	SCGE	10, 50, 100 and 200 mM	24 h exposure- TL increase at 50, 100 mM (*p* < 0.05). 48 h- TL, TM, TI significant increase at 200 mM (*p* < 0.05)	Milić et al. 2006 [40]
	Ly	SCGE	1–50 µM	A significant (*p* < 0.05) dose-dependent increase in OTM;TL;TI from 2.5 µM (2.593 ± 0.913; 53.960 ± 13.663; 3.887 ± 0.810) up to 50 µM (93.292 ± 18.218; 234.326 ± 14.846; 74.113 ± 13.238) vs. control (1.924 ± 0.722; 44.830 ± 4.943; 3.125 ± 1.007)	Schmid et al. 2007 [41]
	PSG			A significant *p* < 0.05) dose-dependent increase in OTM;TL;TI from 2.5 µM (3.234 ± 1.244; 54.941 ± 11.062; 4.887 ± 1.611) up to 50 µM (26.021 ± 10.922; 118.644 ± 21.685; 31.035 ± 13.406) vs. control (2.239 ± 0.598; 48.273 ± 4.403; 3.658 ± 0.817)	
	TK6	SCGE	0.01–2 µM	A significant dose-dependent increment in OTM from 0.1 µM (*p* < 0.05) up 2 µM (*p* < 0.001)	Guillamet et al. 2008 [42]
Hg nitrate (Hg^2+^)	Ly	SCE	1–30 µM	No significant differences (*p* > 0.05)	Lee et al. 1997 [43]
		EM	30 µM	Significant increase (*p* < 0.05) 3.4 ± 0.6% compaed with 0.4 ± 0.3% in control	

L—human leukocytes; Ly—human lymphocytes; WB—whole blood; WRL-68—human liver cell line; TK6—human lymphoblastoid cell line; U-937 human macrophage cell line; -PSG—parotid salivary gland cells; CBMN—cytokinesis B blocked micronucleus assay; CA—chromosomal aberrations; SCE—sisters chromatid exchange; C—anaphase; MN- micronuclei; 8-OHdG—8-Hydroxy-2’-deoxyguanosine; EM—endoreduplicated mitosis; SCGE—single cell gel electrophoresis or alkaline comet assay with parameters: TL—tail length (µm), TM—tail moment; TI—tail intensity (%); OTM—Olive Tail Moment; Mf—mutant frequency; HPRT—hypoxanthine phosphoribosyltransferase.

**Table 2:** Studies on in-vitro genotoxic effects by organic Hg compounds.

Compound	Cell type	Assay	Concentrations	Results	References
C_2_H_5_HgCl, C_6_H_5_HgCl	HeLa	CA	1.0–1.8 µg/mL	Significant increase	Umeda et al. 1969 [45]
methyl mercury (CH_3_HgX)	Ly	CA	0.05–0.5 ppm	18.7% chromosome breakage, 2.6% chromosome reunions and rearrangements	Kato and Nakamura 1976 [50]
	PB	SCE	8 × 10^−8^–2.5 × 10^−4^ M	Significant increase from 8 × 10^−8^ (10.49 ± 0.55 SCE/cell) up to 2 × 10^−6^ M (12.69 ± 0.60 SCE/cell) vs. control (8.86 ± 0.50) (*p* < 0.05), no cell growth in major concentrations	Morimoto et al. 1982 [30]
	Ly	CA	5–30 µM	Significant increase of chromatid type aberrations from 5 µM (12.87%) up to 30 µM (24%) vs. control (1.00%) and chromosome type aberrations from 5 µM (3.96%) up to 30 µM (16.00%) vs. control (0.00%) (*p* < 0.001)	Verschaeve et al. 1985 [31]
	Ly	CA	0.12–25 µM	Significant increase from 0.6 × 10^−6^ M up to 25 × 10^−6^ M in structural CA (10.00 ± 1.63–23.00 ± 3.46) vs. control (4.50 ± 2.51) and numerical CA (2.50 ± 3.00–10.50 ± 3.41) vs. control (0.00) (*p* < 0.001)	Betti et al. 1992 [46]
	Ly	CA	3–25 µM	Significant increase from 5 µM (6.00 ± 2.82) up to 25 µM (12.00 ± 8.48) (*p* < 0.05) vs. control (0.00)	Betti et al. 1993 [49]
		SCE		Significant increase at 5 µM (7.44 ± 2.44% SCE) and 15 µM (8.04 ± 2.90% SCE) vs. control (5.92 ± 1.84) µM (*p* < 0.05)	
	PB/Ly	CA	1–10 µM	Significant increase at 5 µM (9.3 ± 1.7) and 10 µM (22.3 ± 5.9) vs. control (3.0 ± 0.0)(*p* < 0.01)	Ogura et al. 1996 [33]
		MN		Significant increase of MN in 3000 cells at 5 µM (43) and 10 µM (65) (*p* < 0.01) vs. control (25)	
	PB/Ly	8-OHdG	1–10 µM	The level of 8-OHdG was also significantly (*p* < 0.05) elevated (1.111 ± 0.221; 5 × 10 µM) vs. control (0.373 ± 0.116)	Ogura et al. 1996 [33]
	Ly	SCE	0.3–20 µM	Significant increase at 20 µM (11.4 ± 0.5 SCE/cell) vs. control 7.0 ± 0.4) (*p* < 0.05)	Lee et al. 1997 [43]
		EM	20 µM	Significant increase (*p* < 0.05) 4.2 ± 0.5% compaed with 0.4 ± 0.3% in the control	
	Ly	CA	0.1–1000 μg/L	Significant increment of CA from 13.5% at 0.1 μg/L to 12.2% at 1000 μg/L not dose related and polyploidy from 13.0 ± 1.3546 at 0.1 μg/L to 64.3 ± 1.8961 dose related (*p* < 0.5)	Silva-Pereira et al. 2005 [38]
	U373	CBMN	0.1 and 1 μM	Significant increase between 11–12% in the frequency of micronucleated cells (*p* < 0.05)	Crespo-López et al. 2007 [6]
	B103			Non-significant increase in frequency of MN cells between 6–8% in the frequency of micronucleated cells (*p* > 0.05)	
	TK6	SCGA	0.01–3 μM	Significant increment inn OTM (*p* < 0.001) at 3 μM	Guillamet et al. 2008 [42]
	PB	MN, CA	1–500 μg/L or 0.004–2 μM	Loss of cells proliferative capacity, very low frequency of MN (0.3 at 1, 10 and 50 μg/L), no correlation with Hg concentration, no CA	Crespo-López et al. 2011 [51]
	C6	SCGE, CBMN	3 μM	Significant increase of TI, MN and NA (*p* < 0.01)	Crespo-López et al. 2016 [52]
	SH-SY5Y	SCGE	3–30 mg/L CH_3_HgCl	Significant increase of fragmentation index from 7 ± 2.64% at 3 mg/L up to 98.6 ± 0.57% at 30 mg/L and TL from 1.6 ± 0.25 µm at 3 mg/L up to 32.8 ± 1.53 µm at 30 mg/L	Patnaik and Padhy 2018 [53]
			3–42 mg/L CH_3_HgOH	Significant increase of fragmentation index from 3 ± 1.73% at 3 mg/L up 98 ± 0.57% at 30 mg/L and TL from 2.2 ± 0.95 µm at 3 mg/L up to 20.4 ± 0.77 µm at 30 mg/L	
[(CH_3_)_2_Hg]	Ly	CA	0.34–434 µM	Significant increase in structural CA at 43.4 × 10^−6^ M (9.00 ± 2.58), 217 × 10^−6^ M (9.50 ± 3.00 and 434 × 10^−6^ M (12.00 ± 2.82) vs. control (4.50 ± 2.51) and numerical CA from 1.73 (2.50 ± 1.00) up to 434 (5 ± 2) vs. control (0.00) (*p* < 0.05)	Betti et al. 1992 [46]
PMA	Ly	SCE	1–30 µM	Significant SCE increase from 10 µM (9.5 ± 0.4 SCE/cell) up to 30 (14.9 ± 0.6) µM vs. control (7.0 ± 0.4) (*p* < 0.05)	Lee et al. 1997 [43]
		EM		Significant increase from 3 µM (3.1 ± 0.7) up to 30 (15.2 ± 0.9) µM vs. control (0.4 ± 0.3) (*p* < 0.05)	
thiomersal	Ly	CBMN	0.05 and 0.6 µg/mL	Significant induction (*p* < 0.05) was seen at concentrations between 0.05–0.5 µg/mL in 14 out of 16 experiments, with individual and intraindividual variations among the different donors.	Westphal et al. 2003 [3]
	Ly	SCE, ±S9 metabolic activation	0.2–0.6 µg/mL	Significant SCE induction (*p* < 0.001) between 0.2 and 0.6 µg/mL compared with negative control. A significant decrease (*p* < 0.001) in MI and PRI compared with control cultures	Eke and Celik 2008 [47]

HeLa—human cervix epithelioid carcinoma cell line; MN- micronuclei; CBMN—cytokinesis blocked micronucleus assay; SH-SY5Y—human neuroblastoma cell line; C6—glioma cell line; U373—glioblastoma; B103—neuroblastoma; TK6—human lymphoblastoid cell line; PB—peripheral blood; Ly—human lymphocytes; CA—chromosomal aberrations; SCE—sister chromatid exchange; 8-OHdG—8-Hydroxy-2’-deoxyguanosine; EM- endoreduplicated mitosis; E—endoreduplication; SCGE—single cell gel electrophoresis or comet assay; PMA—phenylmercury acetate; C_2_H_5_HgCl—ethylmercury chloride; C_6_H_5_HgCl—Phenylmercury chloride or PMA; [(CH_3_)_2_Hg]—Dimethylmercury; TL-tail length; TI-tail intensity; TM-tail moment; OTM—Olive Tail Moment; MI- mitotic index; PRI—proliferation index.

**Table 3:** Genotoxic effects in human populations exposed to Hg compounds.

Compound	Cell Type/Assay	Exposure Biomarker	Origin of Hg	E/C (*N*)	Results	Country	Reference
methylmercury (CH_3_Hg)	Ly/CA	Hg levels in RBC	dietary contaminantes fish	9/4	CA-Hg conc significant correlation	Sweden	Skerfving et al. 1970 [54]
	Ly/CA	Hg levels in BC	dietary contaminantes fish	23/16	CA-Hg conc significant correlation	Sweden	Skerfving et al. 1974 [55]
	PB/SCE, CA	Hg hair and PB levels	dietary contaminantes fish	16/14	No significant correlation of Hg hair levels and structural CA or SCE	Colombia	Monsalve and Chiappe 1987 [56]
	PB Ly/cytogenetic damage	Hg hair levels	Tapajós River	98 adults	CH_3_Hg contamination correlates with cytogenetic damage	Brazil	Amorim et al. 2000 [2]
iHg	Buccal cells/MN	Hg urine levels	artisanal and small-scale mining	83 workers	18.1% of exposed people had elevated MN levels	Perú	Rosales-Rimanche et al. 2013 [57]
mHg, amalgams C_6_H_5_Hg, C_2_H_5_Hg+	WB Ly/CA	Hg blood and urine levels	Hg intoxication (10) and accidental exposure (18)	28/7	Significant blood and urine Hg correlation; and both with total amount of cells with CA	Belgium	Verschaeve et al. 1976 [58]
CH_3_COOHgC_6_H_5_	Ly/CA	Hg blood levels	PMA exposure	16/12	significant increase in hyperploidy	Belgium	Verschaeve et al. 1978 [59]
	PB/SCE	Diapers interruption lapse: 9, >9 months	diapers	38	Significant increase (*p* < 0.001)	Argentina	Mudry de Pargament et al. 1987 [60]
mHg	L/CA	Hg urine levels	chloralkali plant	28/20	No significant correlation	Belgium	Verschaeve et al. 1979 [61]
	PB Ly/CA	Hg blood and urine levels	hg-Zn amalgamation and chloralkali plants	22/25	No increase in structural CA	Belgium	Mabille et al. 1984 [62]
	PB Ly/SCE, SCGE	Hg blood and urine levels	chlorine production department	25/50	Not significant difference between workers and controls	Poland	Cebulska-Wasilewska et al. 2005 [4]
	WB/SCGE	Hg blood levels	gold mining	61/51	Significant Hg and damage association	Colombia	Calao and Marrugo 2015 [63]
mHg, oHg	WB/CA	Hg urine levels	chemical plant	22/10	CA was significantly higher	Switzerland	Popescu et al. 1979 [64]
oHg	WB/SCE	Hg blood levels	seal diet	147	Significant Hg and SCE correlation	Greenland	Wulf et al. 1986 [65]
elemental Hg, iHg	Blood/SCE	Hg blood levels	caustic soda, copper sheets plants	29/26	Significant Hg and SCE correlation	United States	Mottironi et al. 1986 [66]
Hg vapor	PB/CBMN	Hg urine, plasma, erythrocytes levels	chloralkali plant	26/26	No correlation between current Hg level and MN	Sweden	Barregård et al. 1991 [67]
	WB/CA and MN	Hg blood and urine levels	chloralkali plant	29/29	No significant differences in CA and MN frequencies.	Norway	Hansteen et al. 1993 [68]
	Ly/MN, SCE and HGPRT	HG urine level	chloralkali industry	30/30	Higher levels of MN, SCE, and HGPRT mutations	Egypt	Shamy et al. 1995 [69]
	WB Ly/MN	Hg urine levels	mercury producing plant	15/15	Significant increase of MN frequency	Brazil	Queiroz et al. 1999 [70]
	Ly/CA	Hg levels in the air	stomatological cabinets	40/24	Significan increase with 7 or more years of exposure	Lithuania	Lazutka et al. 1999 [71]
			battery plant	114/26			
	WB/MN and SCE	Hg blood levels	river silt aerosols	100/100	No significant differences in MN and SCE frequencies.	Germany	Wegner et al. 2004 [72]
Hg fulminate	WB/CBMN, CA	Hg urine levels	explosives factory	29/29	Significant increase, no correlation with exposure duration nor Hg urine level	Egypt	Anwar y Gabal 1991 [73]
iHg	WB/MN, CA, SCE	-	mercury mining	10/10	Significant increase	Slovenia	Al-Sabti et al. 1992 [74]
oHg	PB Ly/MN	Hg blood levels	contaminated seafood	51 fishermen	Significant correlation of MN frequency and total Hg in blood	Italy	Franchi et al. 1994 [75]
iHg	uroepithelial cells/MN, NA	Hg urine levels	mining zone	104 females	Possible association between cytogenotoxicity and Hg level	Mexico	Soto-Ríos et al. 2010 [76]
	blood/CBMN	Hg blood levels	environment	110 newborns 136 pregnant, 134 fathers	Elevated blood Hg levels in fathers were associated with significantly higher MN	Madrid, Spain	Lope et al. 2010 [77]
	blood/SCGE	Hg blood levels	mining sites	50/50	Statistical significant increase	Colombia	Cruz-Esquivel et al. 2019 [78]
	oral mucosa cells/MN, NA						
amalgam	Ly/CA	-	dentistry	10 /10	Statistical significant increase	Belgium	Verschaeve and Susanne 1979 [79]
	Ly/SCGE	-	dental restaurative fillings	44/24	Association between dental fillings and DNA damage	Italy	Di Pietro et al. 2008 [80]
	Buccal cells/ SCGE, MN	-	dental restaurative fillings	63	Association between dental fillings and DNA damage	Italy	Visalli et al. 2013 [81]
	WB/SCGE	Hg urine levels	gold mining and burners	32/32	Greater genetic damage in those exposed than in controls	Colombia	Castaño Arias et al. 2014 [82]
	Buccal cells/MN	-	dental restaurative fillings	110	Increase of genotoxic effect with dental fillings	India	Mary et al. 2018 [83]

Ly—human lymphocytes; CA—chromosomal aberrations; MN—micronuclei; WB—whole blood; C_6_H_5_Hg—phenylmercury; C_2_H_5_Hg^+^—ethylmercury; L—human leukocytes; PB—peripheral blood; CBMN—cytokinesis blocked micronucleus assay including not only MN but other biomarkers; SCE—sisters chromatid exchange; 8-OHdG—8-Hydroxy-2’-deoxyguanosine; (CH_3_)_2_Hg—dimethylmercury; iHg—inorganic mercury; mHg—metallic mercury; oHg—organic mercury; CH_3_COOHgC_6_H_5_—phenylmercury acetate; Contam water—contaminated water; SCGE—single cell gel electrophoresis or alkaline comet assay; ROS—reactive oxygen species; MI- mitotic index; BC—blood cells RBC-red BC; conc-concentration; E—exposed; C—control.

## Data Availability

Not applicable.

## Supplementary Materials

The following are available online at https://www.mdpi.com/article/10.3390/toxics9120326/s1. Table S1: PRISMA 2020 Main Checklist; Table S2: PRIMSA Abstract Checklist; Figure S1: PRISMA 2020 flow diagram for Hg systematic reviews including searches of databases and registers only.
